# Localized Delayed T-cell Mediated Hypersensitivity After mRNA SARS-CoV-2 Vaccination

**DOI:** 10.7759/cureus.35271

**Published:** 2023-02-21

**Authors:** Nikita Jhawar, Juan Cardenas Rosales, Alexei Gonzalez-Estrada

**Affiliations:** 1 Internal Medicine, Mayo Clinic, Jacksonville, USA; 2 Allergy/Immunology, Mayo Clinic, Phoenix, USA

**Keywords:** covid-19 vaccination, vaccination, sars-cov-2, exanthem, allergy, hypersensitivity

## Abstract

Since the emergency authorization of SARS-CoV-2 vaccines, the medical literature has been investigating the management of allergic reactions to the vaccines. Anaphylaxis has been reported among a minority of vaccinated individuals, and many trials monitoring the safety profile of the vaccines have identified cases of benign cutaneous reactions. Typical features of delayed benign cutaneous hypersensitivity reactions include localized erythema, pruritus, and rash. However, reports have described rare cases of rash and atopy at sites apart from the injection site following vaccine delivery. We will discuss a unique case of delayed benign cutaneous hypersensitivity presenting in the lower extremity after an upper-extremity administration of an mRNA SARS-CoV-2. Additionally, we describe management strategies to guide clinicians who encounter similar vaccine-induced hypersensitivity reactions.

## Introduction

Clinical trials investigating the safety profile of mRNA SARS-CoV-2 vaccines have demonstrated rare cases of anaphylaxis and some cases of delayed cutaneous reactions. Per the Centers for Disease Control and Prevention, the rate of anaphylaxis for the Moderna mRNA SARS-CoV-2 vaccine and the Pfizer mRNA SARS-CoV-2 vaccine were 2.5 cases per million and 11.1 cases per million, respectively [1]. After much deliberation about allergic reactions being contraindications to subsequent vaccinations, the global expert consensus is that vaccine benefits supersede the risks associated with developing COVID-19 infection. Patients with anaphylaxis to prior SARS-CoV-2 vaccines may receive future boosters under the observation of an allergist in a facility where anaphylaxis can be recognized and treated. We discuss a case of delayed cutaneous benign hypersensitivity in a patient who received a dose of the Pfizer mRNA SARS-CoV-2 vaccine and recommendations for future management as well as subsequent vaccinations.

## Case presentation

**Figure 1:**
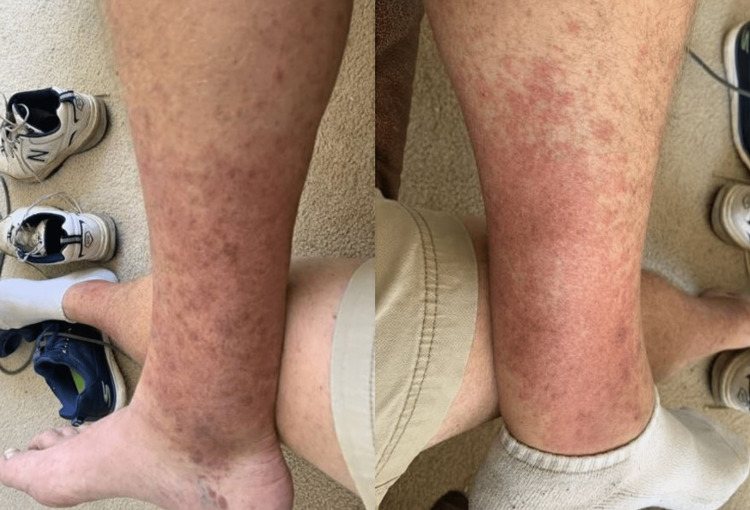
Maculopapular rash of bilateral lower extremities.

A 63-year-old male with no relevant past medical history presented for evaluation of a rash after receiving the first dose of the Pfizer mRNA vaccine against SARS-CoV-2. Of note, the patient denied a known history of SARS-CoV-2 infection before vaccination. Additionally, he denied a prior history of atopy, including seasonal allergies or drug-related hypersensitivity reactions. He also denied having a history of atopic dermatitis, psoriasis, shingles, or any autoimmune conditions like systemic lupus erythematosus. He received the vaccination in his left deltoid without any acute symptoms. Five days after vaccination, the patient developed a fever that lasted for three days and a pruritic, maculopapular rash on his ankles, which spread to his bilateral lower extremities, and eventually to the upper extremities and face (Figure 1). Oral corticosteroid and diphenhydramine therapy resolved the rash.

## Discussion

The patient was evaluated in the clinic for guidance on second-dose vaccination. Although his cutaneous allergy symptoms were not localized to the site of injection in his left deltoid, they are still suggestive of delayed T-cell mediated hypersensitivity. All in all, we present a unique and rare manifestation of delayed hypersensitivity against the mRNA SARS-CoV-2 vaccine. Numerous studies have documented delayed cutaneous hypersensitivity reactions after mRNA SARS-CoV-2 vaccination. Although most delayed reactions present with hives, injection-site erythema, pruritus, and induration, erythematous lesions appear less commonly on legs, thighs, and forearms [2,3]. On average, symptoms present seven days after receipt of the vaccine, with an average symptom duration of five days [3].

Retrospective case series have demonstrated that these reactions are mostly self-limited without serious adverse effects [3]. Supportive care with antihistamines and steroids may be utilized for symptom management [4]. Additionally, delayed reactions are not contraindications to receiving further SARS-CoV-2 mRNA vaccinations. Individuals who experienced an immediate allergic reaction to the first dose of the vaccine are recommended to receive the second dose in a setting equipped to recognize and manage the symptoms of anaphylaxis [3]. For future vaccinations or boosters, H1-antihistamine or oral corticosteroids can be prescribed before vaccination to temporarily diminish the immune response elicited by vaccination [5]. Our patient received his second vaccination with prophylactic cetirizine one month after the first without adverse reactions.

In Moderna’s mRNA SARS-CoV-2 vaccine clinical trial, 244 subjects (0.8%) experienced delayed cutaneous allergic reactions after the first dose, and 68 subjects (0.2%) experienced them after the second dose [6]. Among, all the hypersensitivity reactions reported in the trial, delayed cutaneous reactions were the most reported [6,7]. However, there are uncommon cases of delayed cutaneous reactions occurring at sites aside from the injection site [6]. In one such case, a 55-year-old patient who received the vaccination in the left deltoid developed pruritic papules and erythematous lesions diffusely on her body [8]. For both mRNA vaccine preparations, several different excipients may play a role in hypersensitivity reactions, including polyethylene glycol, polysorbate 80, adjuvants, unintentional contaminants, and other preservatives [9]. Hypersensitivity reactions against polyethylene glycol have been reported in immediate hypersensitivity reactions but are not well-delineated for delayed-type hypersensitivity [3]. The underlying mechanism driving hypersensitivity reactions is hypothesized to be T-cell mediated against one of the aforementioned vaccine components [3]. Although there are no valid testing protocols in place to ascertain the cause of hypersensitivities to these vaccines, patch testing may be considered [7]. Benign exanthems after SARS-CoV-2 vaccinations, however, are not contraindications for future vaccinations.

## Conclusions

Delayed benign cutaneous hypersensitivity reactions have been reported in several patients receiving mRNA SARS-CoV-2 vaccinations with a median time of onset of seven days. Culprits for these reactions may include, but are not limited to, preservatives, adjuvants, accidental contaminants, and stabilizers. Medical literature suggests that these reactions tend to resolve spontaneously without long-term sequelae. Patients who experience such allergic reactions to the first dose of an mRNA SARS-CoV-2 vaccine may receive subsequent doses or boosters in a setting equipped to manage anaphylaxis, as delayed benign hypersensitivity reactions do not contraindicate patients from receiving further vaccinations or boosters. Individuals with known prior delayed benign hypersensitivity reactions may be pre-medicated with antihistamines for subsequent vaccinations. Additionally, patch testing or delayed intradermal testing may be pursued to ascertain the causative agents implicated in hypersensitivity reactions and to guide further vaccinations, but there is currently no valid testing strategy. 
